# Equitable access to mental healthcare integrated in primary care for people with severe mental disorders in rural Ethiopia: a community-based cross-sectional study

**DOI:** 10.1186/s13033-019-0332-5

**Published:** 2019-12-28

**Authors:** Maji Hailemariam, Abebaw Fekadu, Girmay Medhin, Martin Prince, Charlotte Hanlon

**Affiliations:** 1 Department of Psychiatry, School of Medicine, College of Health Sciences, Addis Ababa University, WHO Collaborating Centre for Mental Health Research and Capacity-Building, Addis Ababa, Ethiopia; 2 Centre for Innovative Drug Development and Therapeutic Trials for Africa (CDT-Africa), College of Health Sciences, Addis Ababa University, Addis Ababa, Ethiopia; 3 Centre for Affective Disorders, Department of Psychological Medicine, Institute of Psychiatry, Psychology and Neuroscience, King’s College London, London, UK; 4 Aklilu Lemma Institute of Pathobiology, Addis Ababa University, Addis Ababa, Ethiopia; 5 Centre for Global Mental Health, Health Services and Population Research Department, Institute of Psychiatry, Psychology and Neuroscience, King’s College London, London, UK

**Keywords:** Mental health services, Rural residence, Mental health, Coverage, Sub-Saharan Africa, Healthcare disparities, Health equity

## Abstract

**Background:**

Integration of mental healthcare into non-specialist settings is advocated to expand access to care for people with severe mental disorders (SMD) in low-income countries. However, the impact upon equitable access for disenfranchised members of society has not been investigated. The purpose of this study was to (1) estimate contact coverage for SMD of a new service in primary healthcare (PHC) in a rural Ethiopian district, and (2) investigate equity of access for rural residents, women, people with physical impairments and people of low socio-economic status.

**Methods:**

Community key informants were trained to identify and refer people with probable SMD in Sodo district, south-central Ethiopia, using vignettes of typical presentations. Records of those referred to the new PHC-based service were linked to healthcare records to identify people who engaged with care and non-engagers over a 6 month period. Standardised interviews by psychiatric nurses were used to confirm the diagnosis in those attending PHC. Non-engagers were visited in their homes and administered the Psychosis Symptom Questionnaire. Socio-economic status, discrimination, disability, substance use, social support and distance to the nearest health facility were measured.

**Results:**

Contact coverage for the new service was estimated to be 81.3% (300 engaged out of 369 probable cases of SMD identified). Reimbursement for transport and time may have elevated coverage estimates. In the fully adjusted multivariable model, rural residents had 3.81 increased odds (95% CI 1.22, 11.89) of not accessing care, in part due to geographical distance from the health facility (odds ratio 3.37 (1.12, 10.12)) for people living more than 180 min away. There was no association with lower socioeconomic status, female gender or physical impairment. Higher levels of functional impairment were associated with increased odds of engagement. Amongst non-engagers, the most frequently endorsed barriers were thinking the problem would get better by itself and concerns about the cost of treatment.

**Conclusion:**

Integrating mental healthcare into primary care can achieve high levels of coverage in a rural African setting, which is equitable with respect to gender and socio-economic status. Service outreach into the community may be needed to achieve better contact coverage for rural residents.

## Background

Despite the substantial disability associated with mental and neurological disorders in most low- and middle-income countries (LMICs), mental health services remain numerically limited, geographically centralised and structurally hospital-based. As a consequence, there is a large treatment gap, with over 90% of people with severe mental disorders (SMD; referring to psychotic and affective disorders associated with recurrent or enduring disability) never receiving evidence-based care in most low-income countries [1]. Inadequate care for people with SMD in LMICs contributes to disability, poverty, marginalisation, premature mortality and human rights abuses [2].

To narrow this large treatment gap, the World Health Organisation (WHO) has introduced the mental health Gap Action Programme (mhGAP) which seeks to expand access to mental healthcare through integration into primary care and general healthcare services [3]. Although the integration approach is conceptually appealing, and has potential to also improve physical healthcare of people with SMD and reduce stigma, actual implementation has been challenging [4]. In response, the PRogramme for Improving Mental health carE (PRIME) was established to investigate effective implementation strategies for integrated care across five LMICs: Ethiopia, India, Nepal, South Africa and Uganda [5, 6].

An important focus of PRIME is to evaluate and address the factors affecting equitable access to integrated mental healthcare for disenfranchised sections of society. In a qualitative study conducted in the Ethiopian intervention site prior to introduction of the new service, participants anticipated that women, people residing in rural areas, those of low socioeconomic status and those with physical and sensory impairments would be less able to access primary healthcare (PHC) based mental healthcare [7]. However, the relevance of these factors once the service has actually been made available is not known. There is well-established evidence that availability of services cannot be equated with accessibility of services for particular segments of society [8–10], including studies from Ethiopia [11, 12]. Older age, being unmarried and lower education levels [12], larger household size, not having a history of previous access to care and lack of media exposure [13], have all been shown to be associated with lower access to general healthcare. Hence, a focus on social determinants is central to understanding barriers to access and designing action towards their alleviation [14, 15].

Most studies of access to mental healthcare in LMICs focus on centralised, specialist services [16], and we are not aware of any published studies that have examined access to integrated primary mental health care services. People with SMD may experience specific barriers to accessing care, even when locally available, due to lack of autonomy, stigma, impoverishment, discrimination and disempowerment related to long-term illness.

The purpose of this study was to (1) estimate contact coverage for people with SMD of a new integrated primary mental healthcare service in a rural Ethiopian district, and (2) investigate equity of access for rural residents, women, people with physical and sensory impairments and people of low socio-economic status.

## Methods

### Study design

Cross-sectional, community-based study.

### Study setting

The study was conducted in Sodo district, one of the 15 districts of the Gurage zone, in the Southern Nations, Nationalities and Peoples’ Regional state of Ethiopia. Sodo is located about 100 km from Addis Ababa, the capital of Ethiopia. Sodo district combines the rural–urban and highland-lowland geography which is representative of the country [5]. The population of the district was estimated to be around 160,000 at the time of the study [17]. The settlement patterns are mostly rural, with only 10% of people living in urban centres [17]. The district has one asphalt road linking it to the neighbouring Oromia region in the North and another Gurage district in the South. The urban settlements largely follow the main road. The district has eight health centres, which serve an average of 40,000 people in urban sub-districts and 25,000 people in rural areas. The health centres are each linked to approximately five health posts run by health extension workers (HEWs; female community health workers with 1 year of training in preventive care after completing grade 10). Prior to the introduction of mental healthcare within all eight health centres through PRIME, the nearest specialist mental healthcare facility was a psychiatric nurse-led out-patient unit located around 30 to 50 km away, or in-patient services in Addis Ababa. Part of the PRIME intervention involved 9 days of training for non-specialist PHC staff (mainly public health officers and nurses) to equip them to deliver mhGAP evidence-based packages of care, including diagnosis, assessment and management of selected priority disorders (SMD, depression, epilepsy and alcohol use disorders) [5].

### Case ascertainment

Sixty-five health extension workers and 26 key informants (mainly project data collectors who are also members of the community and community leaders) were trained to detect people with probable SMD. The training made use of case vignettes that illustrated common presentations of SMDs in this setting. Key informant detection of people with SMD has been shown to be sensitive, compared to house-to-house screening, in a previous study conducted in the neighbouring district [18]. Psychiatrists and supervised psychiatric nurses provided the half-day training. Those who received the training were requested to conduct house-to-house visits to identify people who had symptoms/behaviours that corresponded to the description of mental disorders provided during the training. People identified as having probable SMD were encouraged to attend their local health centre on a designated date. Out of people who attended the health centre, those diagnosed by mhGAP-trained PHC workers as having SMD (diagnoses of psychosis or bipolar disorder according to the mhGAP intervention guidelines) were then assessed by project psychiatric nurses using a standardized, semi-structured diagnostic interview schedule [19]. Where the PHC staff and the psychiatric nurses disagreed, diagnosis by the psychiatric nurses took precedence. People who were confirmed to have SMD, including primary psychotic disorders (schizophrenia, schizoaffective disorder) or affective disorders (bipolar disorder or severe depression/depression with psychotic features), were invited to participate in the study. Six months post-implementation of the new integrated primary mental healthcare service, lists of referrals made by the HEWs and key informants were collected from each sub-district (*kebele*). The referral lists were checked against the registry of those receiving treatment at the health centres. Follow-up visits were carried out to the houses of people who were identified as having probable SMD and referred to PHC but who had not accessed care by the end of 6 months. Both the PRIME project and the PHC registers were reviewed to identify cases who had not been identified originally by community informants, and who might have been eligible for care during the study period.

### Instruments

Access to care was measured by reviewing attendance of people referred to PHC with probable SMD at 6 months after the introduction of the service.

### Measures in non-engaged group

The five item, lay-interviewer administered Psychosis Screening Questionnaire (PSQ) [20], was used to determine presence of probable psychotic symptoms in those who were referred but who did not attend PHCs. Barriers that ever stopped, delayed or discouraged access to mental health care were measured using the Barriers to Access to Care Evaluation (BACE-3) which included a 12 item treatment stigma sub-scale [21]. The Short Explanatory Model Interview (SEMI) [22] was used to elicit causal attributions.

### Measures in engaged group

People who attended PHC-based care underwent a confirmatory diagnostic interview using the Operational Criteria Checklist (OPCRIT) [19] by psychiatric nurses.

### Measures in both engaged and disengaged groups

The WHO 12 item Disability Assessment Schedule (WHODAS 2.0) [23] was used as a continuous measure of disability associated with mental disorders. The WHODAS has been validated for use in people with SMD in rural Ethiopia [24]. Social support was measured using the three item Oslo-3 scale, which measures the extent of the person’s support network, interest shown by others and ease of obtaining practical help [25]. The presence of alcohol use disorder was measured using the Alcohol Use Disorders Identification Test (AUDIT) [26]. A cut-off score of ≥ 8 was used to define presence of alcohol use disorder. Experience of discrimination due to mental illness was measured using the discrimination and stigma scale (DISC-12), using a sub-scale measuring the scope and content of experienced discrimination [27]. To assess physical and sensory impairment, the Brief Physical Impairment Rating Checklist (BPIRC) was used, which was constructed from items from the Washington Group’s disability measure and Work, Family and Well-being (WFW) scales [28, 29]. Constituent items of the scale evaluate physical disability in terms of mobility, ability to use hands, vision, hearing with or without aids and speaking.

### Data collection

Data were collected with structured questionnaires administered by trained lay interviewers whose educational level ranged from 10th grade to a college degree. Supervisors, with educational levels ranging from a diploma to college degree, were also trained and supported by the study investigators throughout the process. Both the data collectors and the supervisors were trained for a week on administering interviews to people with SMD and their caregivers. Their training sessions also involved practice interviews followed by feedback from the group and the trainers. All of the data collectors are residents of the district and thus are familiar with the study setting.

All interview questionnaires were translated into Amharic and translated back to English to evaluate their semantic validity. A team of experts involving psychiatric nurses, psychiatrists, social workers, HEWs, data collectors and field supervisors reviewed the data collection instruments. The review involved attention to language issues and cultural appropriateness of the wording. Comments and concerns raised during the expert consensus meeting were addressed prior to piloting the instruments. For people who accessed care, data were collected at the PHCs after they had seen a healthcare provider. At that time they were given a modest financial reimbursement of 100 birr (approximately $4.5) for transport and time costs. During the referral, none of the people referred with probable SMD were notified about the availability of a reimbursement. For those who were referred but who did not access care, data collectors made home visits.

### Data analysis

Double-data entry was carried out using EpiData version 3.1 and exported to STATA version 14 [30] for analysis.

Contact coverage of mental healthcare was calculated as the number of people with SMD in the district who accessed the integrated mental health service in PHC divided by those who were in need of the service [31].

Open-ended responses on the SEMI were coded and categorised using a coding sheet which had been contextualised for Ethiopia previously. The univariate association of access to care at baseline and the categorical primary exposure variables (gender, residence and physical or sensory impairment), was assessed using the Pearson Chi-square test. For normally distributed variables (poverty index, age, total WHODAS score), Student’s t-test was used. Kruskal–Wallis H test was employed for continuous variables that were not normally distributed (total DISC-12 score). Multiple logistic regression was used to examine factors associated with not accessing mental health care, including the following pre-specified variables: residence, poverty, sex, presence of physical or sensory impairment, age, marital status, educational level, alcohol use disorder, social support, disability related to mental disorders, and discrimination. Crude and adjusted odds ratios with their corresponding 95% confidence intervals were reported. To explore the potential role of geographical distance from the health facility in explaining the association with rural residence, the multivariable model was rerun replacing rural residence with distance from PHC.

### Ethical considerations

The study was approved by the Institutional Review Board of the College of Health Sciences of Addis Ababa University. The Amharic version of the information sheet was read to all of the study participants. Eligible study participants were asked to give their written informed consent or a finger print if they were not literate. Guardian/caregiver permissions were obtained to interview people who lacked capacity to give an informed consent because of SMD. Participants in need of medical attention were referred to the nearest PHC.

## Results

### Contact coverage for SMD

**Fig. 1:**
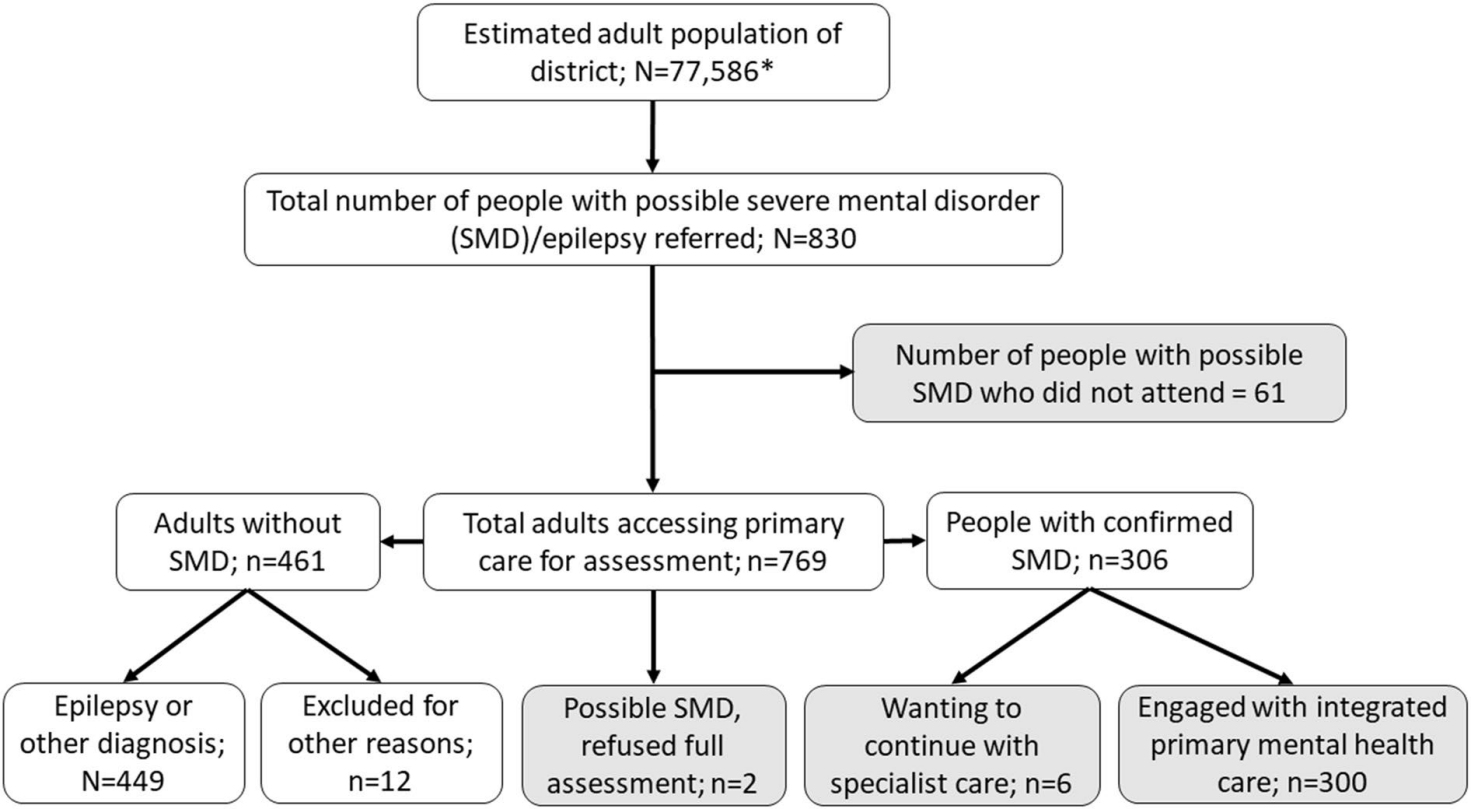
Flow chart of study participants. *Assuming 54% of the total district population to be adults. Greyscale indicates the denominator population of people with probable SMD in the population

A total of 300 people were confirmed to have SMD by the psychiatric nurses. See Fig. 1 for a flow chart of identification, referral, attendance and confirmation of SMD. Two people with possible SMD refused to participate in project assessments. Six people with established diagnoses of SMD preferred to continue receiving care from specialist mental health services located in the neighbouring district or Addis Ababa. After 6 months of the new service being available, 61 people referred with probable SMD had not accessed PHC-based care. Twelve months after recruitment was closed, an additional 28 people had received treatment for SMD at different PHCs in the district. These 28 people were confirmed to be incident cases that would not have been eligible for treatment when treatment was introduced. Therefore, the contact coverage for SMD was calculated to be 81.3% (300/369). Of those who accessed the service, 257 (85.7%) had a primary psychotic disorder and 43 (14.3%) had bipolar disorder or major depressive disorder with psychotic features.

### Characteristics of non-attenders

More than three-quarters of non-attenders (76.2%; n = 45) had one probable psychotic symptom on the PSQ (see Table 1). The most frequently reported description of the nature of the condition was in terms of mental illness or symptoms of mental illness (n = 22; 36.0%), supernatural (n = 9; 14.8%) or another type of health problem (n = 9; 14.8%). The most frequently reported causal attributions were “unknown” (n = 12; 19.7%), “worry and anger” (n = 11; 18.0%) and supernatural (n = 9; 14.8%) (Additional file 1).

**Table 1:** Item frequencies for Psychosis Screening Questionnaire in non-attenders with probable SMD (n = 61)

	Psychosis Screening Questionnaire item	Number (%)
Hypomania		
Introductory question	Have there been times when you felt very happy indeed without a break for days on end?	20 (32.8)
First key question	Was there an obvious reason for this?	6 (9.8)
Second key question	Did your relatives or friends think it was strange or complain about it?	8 (13.1)
Thought insertion		
Introductory question	Have you ever felt that your thoughts were directly interfered with or controlled by some outside force or person?	24 (39.3)
First key question	Did this come about in a way that many people would find it hard to believe, e.g. through telepathy?	16 (26.2)
Paranoia		
Introductory question	Have there been times when you felt that people were against you?	27 (44.3)
First key question	Have there been times when you felt that people were deliberately acting to harm you or your interests?	28 (45.9)
Second key question	Have there been times when you felt that a group of people was plotting to cause you serious injury or harm?	26 (43.3)
Strange experiences		
Introductory question	Have there been times when you felt that something strange was going on?	24 (43.3)
First key question	Did you feel it was so strange that other people would find it very hard to believe?	19 (31.2)
Hallucinations		
Introductory question	Have there been times when you heard or saw things that other people couldn’t?	26 (43.3)
First key question	Did you at any time hear voices saying quite a few words or sentences when there was no one around who might account for it?	28 (46.7)
	Any psychotic symptom	45 (76.4)
	Yes to any introductory question	41 (68.3)
	Yes to first key question	38 (64.4)
	Yes to key question(s) highest level^a^	37 (61.7)

^a^Second key question for hypomania or paranoia, or first key question for the other symptoms

The most frequently endorsed barriers to attending PHC for mental healthcare were the belief that the condition would get better without intervention (44.6%), concerns about the cost of treatment (44.6%) and transport (37.0%), and believing no treatment was available (36.7%). Concerns about stigma were less frequently endorsed (see Table 2).

**Table 2:** Barriers to access to care (BACE) [21] in non-attenders (n = 61)

Barriers to accessing care	Number (%)
Thought would get better by itself	27 (44.6)
Concerns about cost of treatment	27 (44.6)
Lack of money for transportation	23 (37.0)
Thought no treatment for problem	22 (36.7)
Wanted to handle it by themselves	18 (29.1)
Problem did not bother them	18 (29.1)
Unsure about where to go or who to see	12 (19.7)
Concerns about time	10 (16.9)
Treatment did not work before	9 (14.5)
Ashamed to seek help	8 (13.1)
Scared of forced hospitalisation	7 (11.8)
Not satisfied with available care	7 (11.8)
Thought people might look down upon them	7 (11.8)
Worried people might find out	6 (9.4)
Worried about side-effects of treatment	5 (8.0)
Worried about being treated differently	5 (8.0)
Concerns that might harm chances of work	5 (8.0)
Childcare and logistic problems	3 (4.2)
Concern that might harm chances of marriage	3 (4.2)
Thought family would resist	1 (1.4)

### Comparing attenders and non-attenders

Rural residence, but not gender, socio-economic status or physical/sensory impairment, was significantly associated with non-attendance for care (see Tables 3 and 4). In the multivariable model, people with probable SMD who did not attend the PHC-based mental healthcare were more likely to reside rurally and to have less mental disorder-related disability than those who accessed services. When the multivariable model was rerun with ‘rural residence’ removed, living more than 180 min walking distance away from PHC was associated significantly with not accessing care.

**Table 3:** Characteristics of study participants

Characteristic	Accessed primary care-based mental health care (n = 300) Number (%)	Did not access mental health care (n = 61) Number (%)	Statistical significance	
			Test statistic (degrees of freedom)	P-value
Socio-demographic				
Age (years)				
18–24	65 (21.7)	13 (21.1)	z − 0.78 (1)	0.44^b^
25–34	80 (26.7)	23 (38.0)		
35–44	79 (26.3)	12 (19.7)		
45–59	55 (18.3)	8 (13.1)		
60+	21 (7.0)	5 (8.0)		
Gender				
Male	173 (57.4)	37 (60.6)	χ^2^ 0.21 (1)	0.64
Female	128 (42.5)	24 (39.3)		
Residence				
Urban	60 (20.0)	4 (6.6)	χ^2^ 6.28 (1)	0.01
Rural	240 (80.0)	57 (93.4)		
Marital status				
Married	102 (33.8)	17 (27.8)	χ^2^ 0.83 (1)	0.36
Not married	199 (66.1)	44 (72.1)		
Education				
Formal education	144 (47.8)	30 (49.1)	χ^2^ 0.04 (1)	0.85
Non-literate	157 (52.1)	31 (50.8)		
Distance from health centre				
60 min or less	193 (84.2)	36 (15.72)	z 1.26 (1)	0.21^b^
61–120 min	60 (81.0)	14 (18.9)		
121–180 min	36 (90.0)	4 (10.0)		
More than 180 min	11 (61.1)	7 (38.8)		
Religion				
Orthodox Christian	271 (90.3)	57 (93.4)	χ^2^ 1.01 (2)	0.60
Muslim	10 (3.2)	2 (3.8)		
Protestant/other	20 (6.4)	2 (3.8)		
Social support				
Intermediate or good	206 (69.3)	41 (67.2)	χ^2^ 0.11 (1)	0.74
Poor	91 (30.6)	20 (32.7)		
Discrimination (total score on DISC-12)	Median 4 (25th centile 1, 75th centile 8)	Median 3 (25th centile 0, 75th centile 9)	χ^2^ 0.23 (1)	0.63^c^
Poverty index (0 to 8)^a^	Mean 4.2 (SD 1.8)	Mean 4.4 (SD 1.9)	t 0.66 (352)	0.51

Clinical	N (%)	N (%)		
Physical/sensory impairment (“great deal of difficulty”)				
Mobility	26 (8.6)	7 (11.5)	χ^2^ 0.18 (1)	0.68
Using hands or fingers	19 (6.3)	6 (9.8)		
Seeing	4 (1.3)	1 (1.6)		
Hearing	19 (6.3)	1 (1.6)		
Hazardous alcohol use				
AUDIT < 8	221 (73.4)	44 (72.1)	χ^2^ 0.04 (1)	0.84
AUDIT ≥ 8	80 (26.5)	17 (27.8)		
Disability (total score on WHODAS 12)	Mean 35.7 (SD 11.5)	Mean 30.5 (SD 13.6)	t 3.13 (df 356)	< 0.01

*WHODAS 12* World Health Organization Disability assessment Schedule, *SD* standard deviation, *PHQ* Patient Health Questionnaire, *AUDIT* Alcohol Use Disorders Identification Checklist

^a^Poverty index = composite including type of roof, kitchen, having mobile phone, television, radio, access to electricity, sanitation, and source of water

^b^Test for trend

^c^Kruskal–Wallis equality-of-populations rank test

**Table 4:** Multivariable model for factors associated with non-accessing primary care-based mental healthcare (n = 345)

Characteristics	Crude odds ratio (95% confidence interval)	Adjusted odds ratio (95% confidence interval)
Disadvantaged groups		
1 point increase on poverty index^a^	1.05 (0.90, 1.22)	0.98 (0.82, 1.17)
Female sex	0.87 (0.49, 1.53)	0.85 (0.43, 1.65)
Presence of physical or sensory impairment	0.92 (0.63, 1.35)	1.32 (0.54, 3.23)
Rural area residence	3.56 (1.24, 10.21)	3.81 (1.22, 11.89)
Other associated factors		
Age (years)	0.99 (0.97, 1.01)	0.99 (0.96, 1.01)
Unmarried	1.32 (0.72, 2.43)	1.81 (0.84, 3.93)
No formal education	0.94 (0.54, 1.64)	1.20 (0.63, 2.30)
Presence of alcohol use disorder	1.06 (0.57, 1.97)	1.18 (0.55, 2.50)
Poor social support	1.10 (0.61, 1.98)	1.49 (0.78, 2.86)
1 point increase in WHO Disability Assessment Schedule	0.96 (0.94, 0.98)	0.95 (0.92, 0.98)
1 point increase in discrimination and stigma scale	0.99 (0.95, 1.03)	1.01 (0.97, 1.06)

^a^*Poverty index* = composite including type of roof, kitchen, having mobile phone, television, radio, access to electricity, sanitation, and source of water

## Discussion

In this community-based study from Ethiopia, contact coverage with a new service which integrated mental healthcare into primary care was high (81.3%) and equitable with respect to gender, physical and sensory impairment, and socio-economic status. Rural residents had lower odds of accessing the service. Higher levels of mental disorder-related disability were associated with increased odds of engagement with PHC-based mental healthcare. The commonest reasons endorsed by those who did not engage with the service related to expectations that the illness to improve on its own and concerns about costs of accessing care.

### Contact coverage of mental healthcare

A thorough case ascertainment process was used to identify people with probable SMD. The involvement of community-based health extension workers and community key-informants facilitated the tracing and referral of people with probable SMD in the district. We did not identify any cases missed during the initial ascertainment who engaged subsequently. None of the 61 non-engagers were treated for SMD in the following year. All of those newly identified as contacting services (28 people) were reported to have developed the illness after the initial period of case ascertainment. This supports the previous finding that key informants have high sensitivity in detecting people with probable SMD in the context of rural Ethiopia [32].

Epidemiological estimates from neighbouring districts in Ethiopia indicate that schizophrenia and bipolar disorder have a combined prevalence of 0.97% of the adult population [33, 34]. Out of the 160,000 total population of the Sodo district, an estimated 77,586) are adults [17]. If we take a conservative estimate that 50% of people with SMD need ongoing care, this would amount to 423 people needing services in Sodo district (compared to the 369 we identified). The variation of our study findings from this epidemiological estimate could be explained by the under-representation of people with affective psychosis in our sample, with only 14.3% of people with SMD accessing services having an affective psychosis. This may be due to the episodic course of bipolar disorder and better functional recovery between episodes [35]. Therefore, these people are less likely to be identified by our methods of ascertainment.

### Comparing attenders and non-attenders

Rural residence was associated with non-attendance for PHC-based care. Our analyses indicate that geographical distance from the health centre is an important contributor to the effect of rural residence: residing more than 180 min away from PHC was associated significantly with not accessing care. Geographical inaccessibility was also reported by field workers to be a barrier to attending for care. Some sub-districts in Sodo are hilly and do not have all-weather paths or roads. In our qualitative exploration of reasons for not engaging with care, respondents emphasised the difficulty conveying a person with SMD who does not agree to treatment when public transport is not available [36].

Although other studies suggested poorer households could have lesser access to health services [37] and this was anticipated to be a barrier in the formative qualitative work [7], we found that poverty was not a barrier for initial contact with PHC-based mental healthcare. Nevertheless, in those who did not attend, affordability of accessing care was an important concern. A possible explanation is the relative homogeneity of socio-economic status in the study site, and the challenge of distinguishing between levels of poverty. In our follow up qualitative work in this setting, affordability of treatment was reported to be a challenge to ongoing access to care for all participants [36]. Even those who had managed to initiate access to PHC-based mental healthcare doubted their ability to pay for ongoing access. Those who did managed to mobilise funds to attend at least once may have been motivated by the greater severity of disorder, as indicated by the higher level of mental disorder-related disability.

In our study, only 9 (14.8%) people who did not access PHC-based mental healthcare framed their problem in terms of supernatural aetiology. The most frequently provided explanation of the problem in those who did not engage was either mental illness or symptoms of mental illness (n = 22; 36.0%). This could be the effect of social desirability bias as there is an educational level difference between the respondents and the data collectors, even though the data collectors were recruited from the same community. We were not able to compare differences in causal attributions of mental illness between those who did not engage and those who did engage. This could be an important area of exploration for future studies.

### Implications

This study focused on contact coverage with integrated mental health services, but this only reflects one-off contact with services and may not translate into mental health benefits (i.e. effectiveness coverage). There is need for prospective evaluation of the equity of access to long-term care in people with SMD in this setting. As discussed, from our qualitative work [36, 38], we anticipate that poverty will be an important factor in sustained engagement with mental health care.

Addressing the multiple challenges faced by rural residents is key to achieving equitable access to PHC-based mental healthcare in this setting. Interventions to expand access to affordable means of transportation may facilitate engagement by rural residents and people who have to travel long distances to get to the nearest health centre. Moreover, flexible service configurations such as service outreach into the community may be needed to achieve better coverage for rural residents.

Protection of economically vulnerable families against catastrophic healthcare expenditure through a universal health insurance coverage could be helpful. Families often experience unbearable economic distress and may fail to access healthcare due to a system that is structured around out-of-pocket financing [39]. Augmenting the current low-cost healthcare service delivery with other economic interventions such as livelihood diversification could facilitate access by the economically vulnerable. Interventions such as revolving credit services via involvement in service user groups could enable destitute households to engage with care. Strengthening rural literacy programs and introducing mental health awareness raising initiatives could enable people who did not access care due to the perception that mental illness is not treatable.

## Strengths and limitations

As far as we are aware, this is the only community-based study investigating access to primary care-based mental health care for people with SMD in a low-income country. Strengths of the study include the use of validated and contextualised measures, and the involvement of mental health specialists to confirm the diagnosis of SMD. There were, nonetheless, potential limitations of our study. People with SMD who were away from their homes, for example due to migration to larger urban centres or seeking help from traditional and faith-based healing sites, at the time of the study would not have been included.

The study provided reimbursement for travel costs and time to those who attended the PHC on the designated date. Although the potential participants were not informed about this payment by the community key informants, it is possible that some heard by word-of-mouth and that this payment could have acted an incentive to attend the facility. However, the amount of compensation provided for the study participants was modest and not large enough to lead all probable cases to attend PHC.

The lack of association between poverty and non-attendance could reflect the lack of standardized and operational measures of poverty for this setting, although we made use of a diverse indicators of poverty which were informed by measures used in the Demographic and Health Survey for Ethiopia [40, 41]. Due to logistical limitations, we were not able to administer OPCRIT+ to the people with possible SMD who did not engage with care. This would have required specialist mental health professionals travelling to remote rural households which was not feasible. Therefore, it is possible that some of the people who did not engage with care did not have SMD. This would have led us to under-estimate the contact coverage.

## Conclusion

Integrating mental healthcare into primary care can achieve high levels of coverage in a rural African setting, which is equitable with respect to gender, physical impairment and socio-economic status. Flexible service configurations such as service outreach into the community may be needed to achieve better coverage for rural residents.

## Supplementary information

**Additional file 1:** Perceived nature and cause of the problem in non-attenders with probable SMD (n = 61).

## Data Availability

The data are being used for a Ph.D. student (MH) for her thesis and are not, therefore, available at the present time to the general public. The data may be requested from the corresponding author for verification of the analyses in this paper.

## Abbreviations

BACE: Barriers to Access to Care Evaluation
BPIRC: Brief Physical Impairment Rating checklist
DISC: discrimination and stigma scale
HEW: health extension workers
mhGAP: mental health Gap Action Programme
LMIC: low- and middle-income countries
OPCRIT: Operational Criteria Checklist
PRIME: PRogramme for Improving Mental health carE
SEMI: Short Explanatory Model Interview
SMD: severe mental disorders
WHO: World Health Organisation
WHODAS: World Health Organisation Disability Assessment Schedule
WFW: Work and Family Well-being
PHC: primary health care
PSQ: Psychosis Screening Questionnaire
