# Supplementary material for: Equitable access to mental healthcare integrated in primary care for people with severe mental disorders in rural Ethiopia: a community-based cross-sectional study
Source: Int J Ment Health Syst. 2019 Dec 28;13:78. doi: 10.1186/s13033-019-0332-5 (PMC6935213; doi:10.1186/s13033-019-0332-5)
Supplement: Supplementary file 1 — Additional file 1: Perceived nature and cause of the problem in non-attenders with probable SMD (n = 61). [file 13033_2019_332_MOESM1_ESM.doc]

**Additional file 1: Perceived nature and cause of the problem in non-attenders with probable SMD (n=61)**

| **Explanation** | **Perceived type of problem (n)** | **Perceived cause (n)** |
| --- | --- | --- |
| Mental illness | 17 | 0 |
| Behavioural or symptoms of mental illness | 5 | 4 |
| Worry and anger | 7 | 11 |
| Other health conditions | 9 | 6 |
| Alcohol or substance use problems | 2 | 2 |
| Poverty or adverse life events | 6 | 7 |
| Unknown | 5 | 12 |
| Nature | 0 | 1 |
| No problem | 1 | 0 |
| Supernatural | 9 | 9 |
| Interpersonal | 0 | 3 |
